# Trends From 2010 to 2019 in Opioid and Nonopioid Pain Management After Total Knee Arthroplasty

**DOI:** 10.5435/JAAOSGlobal-D-23-00062

**Published:** 2024-06-12

**Authors:** Tyler Bahoravitch, Max Roberts, Amy Zhao, Seth Stake, Brady Ernst, Savyasachi C. Thakkar

**Affiliations:** From the Department of Orthopaedic Surgery, George Washington University School of Medicine and Health Sciences, Washington, DC (Dr. Bahoravitch, Ms. Zhao, and Dr. Stake); the Department of Biochemistry, Georgetown University Medical Center, Washington, DC (Dr. Roberts); the Department of Orthopaedic Surgery, Virginia Commonwealth University School of Medicine, Richmond, VA (Dr. Ernst); and the Department of Orthopaedic Surgery, Johns Hopkins University School of Medicine, Baltimore, MD (Dr. Thakkar).

## Abstract

**Introduction:**

As the opioid epidemic enters its third decade, we reflect on how it has affected clinical practice within the orthopaedic community. Recent studies show prolonged opioid use after total knee arthroplasty (TKA) is associated with worse overall health outcomes. This study aims to elucidate trends in pain management after TKA over the past decade.

**Methods:**

A retrospective analysis was performed using the PearlDiver database from 2010 to 2019. Patients who underwent primary TKA without a history of mental illness, complex pain syndromes, or opioids used 6 months before surgery were selected. Postoperative prescription filling rates of opioid and nonopioid at 30, 90 days, and 1 year from surgery were analyzed. Linear regression analysis and compound annual growth rates (CAGRs) were analyzed from 2010 to 2019, a *P* value <0.05 being considered significant.

**Results:**

Between 2010 and 2019, 579,269 patients underwent primary TKA. At 30 days, filling of prescriptions for opioids (CAGR = 3.54%) and nonopioids (CAGR = 15.50%) markedly increased from 2010 to 2019. At 90 days, opioids decreased (CAGR = −4.42%). At 1 year, opioid (CAGR = −10.92%) and nonopioid (CAGR = −2.12%) prescriptions markedly decreased from 2010 to 2019.

**Discussion:**

This study highlights patterns of decreased opioid prescription rates at 90 days and 1 year postoperatively from 2010 to 2019. Decreasing opioid rates may indicate effectiveness in targeted public health campaigns to curb opioid overuse.

Opioid medications can be effective pain management tools after total knee arthroplasty (TKA). However, since the beginning of the opioid epidemic in the 1990s, the United States has seen a fivefold increase in overdoses, with approximately 75% of drug overdose deaths in 2020 involving opioids.^1^ This is due to a myriad of reasons such as misrepresentation of the addictive nature of opioids, pain becoming the “fifth vital sign,” and pain scores being tied to reimbursement.^2-4^ In response to the growing opioid concern, various medical governing bodies have enacted policies to reduce the volume of prescribed opioids. Notably, orthopaedic surgeons are the third highest prescribers of opioids among physicians in the United States.^5^ To reduce the risk of opioid abuse in patients, the American Academy of Orthopaedic Surgeons implemented their revisions to pain management guidelines in 2015, which included limiting opioid prescription size, extended-release opioids, and preoperative opioid prescriptions. An analysis by Tan et al^6^ found that postoperative patients, between 2014 and 2017, may consume less than half of the medication they are prescribed. A separate study found that one-third of TKA patients have preoperative opioid use within 3 months of surgery, which is a strong predicative indicator of increased opioid prescription refills.^7^ There is also evidence that chronic opioid use is a common occurrence after TKA surgery, especially in patients with a history of psychiatric diagnoses.^8^ It is also known that opioids in the home increases the risk of opioid overdose of others in the home.^9^ This study's aim was to identify trends in opioid prescription filling for patients after TKA. We hypothesized that opioid prescription volume would decrease over the study period.

## Methods

A retrospective trends analysis was conducted using the PearlDiver (Mariner data set) database (www.pearldiverinc.com; 10435 Marble Creek Circle). The Mariner data set contains over 120 million patients and includes all payer claims information from 2010 to 2020. This includes cash pay, private insurance, Medicare, Medicaid, and any other payment form. The PearlDiver database includes records from the United States without geographic limitation and includes all claims regardless of provider type. Patient records were selected using the International Classification of Disease (ICD), Common Procedural Terminology, Uniform System of Classification, and codes specific to PearlDiver. Codes used are provided in Supplemental Appendix (http://links.lww.com/JG9/A343) for reference. This study was institutional review board exempt because the database only permits the extraction of deidentified patient information.

### Selection Criteria

Patients were selected using Common Procedural Terminology, ICD-9, and ICD-10 codes who underwent primary TKA between 2010 and 2019 met initial inclusion criteria. Patients with history of anxiety disorders, mood disorders, personality disorders, psychotic disorders, chronic pain, neoplastic pain, complex regional pain, central pain syndrome, psychosis, dementia, drug abuse, drug dependence, or opioid use 6 months before TKA were excluded in an effort to select patients at lower risk of chronic opioid use.^10,11,12,13^ Follow-up periods of 30 days, 90 days, and 1 year after TKA were chosen to elicit short-term and long-term use of medications. Medications were broken into two categories: (1) opioids and (2) nonopioids. Nonopioids consisted of nonsteroidal anti-inflammatory medications (celecoxib, diclofenac, ibuprofen, ketorolac, meloxicam, and naproxen) and gabapentinoids (gabapentin and pregabalin) with their associated brand names as well. Of note, these claims represent prescriptions filled, including refills, and do not include the total number of prescriptions written or consumption of medications and will be referred to as prescriptions.

### Analysis

Annual prescription prevalence rates (patient records with prescription/total patient records included) were calculated for opioids and nonopioids at 1 to 30 days (30 days), 31 to 90 days (90 days), and 91 to 365 days (1 year). These were calculated for each year (2010 to 2019). Average daily milligram morphine equivalents (MME) were also calculated for the filled prescriptions within the 30- and 90-day periods. Linear regression and CAGR ((Y2/Y1)^1/(Y2 − Y1)^ − 1; Y1 = first year of the analysis, Y2 = final year) were calculated to analyze trends in prescriptions from 2010 to 2015, 2016 to 2019, and 2010 to 2019. The year 2016 was used as a proxy for the implementation of new guidelines. A *P* value of <0.05 was considered significant. PearlDiver does not report patient records with less than 11 claims to protect privacy.

## Results

**Figure 1:**
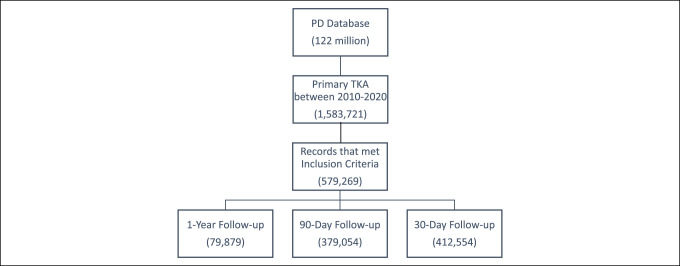
Flowchart of selection of the study population with appropriate follow-up within the PearlDiver database.

In total, 579,269 patients underwent TKA between 2010 and 2019 and met our inclusion criteria. Of these, 412,554 patients had follow-up for 30 days, 379,054 patients had follow-up for 90 days, and 79,879 patients had follow-up for 1 year (Figure 1).

### 30-Day Prescription Claim Rates

**Figure 2:**
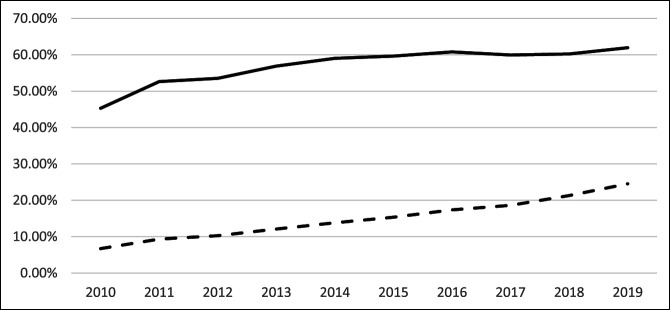
Graph showing annual rates of filled prescriptions for opioids and nonopioids within 30 days of TKA from 2010 to 2019. TKA = total knee arthroplasty

Between 2010 and 2015, prescriptions for opioids (*P* = 0.004) and nonopioids (*P* < 0.001) significantly increased after TKA, with CAGR of 5.56% and 17.98%, respectively. After 2016, only nonopioid prescriptions showed a significant increase (*P* = 0.018; CAGR = 12.21%) while opioid prescriptions showed no significant trends (*P* = 0.489). Overall trends from 2010 to 2019 showed significant increased utilization for opioids (*P* < 0.001; CAGR = 3.54%) and nonopioids (*P* < 0.001; CAGR = 15.50%) (Table 1, Figure 2).

**Table 1:** Annual Prevalence (2010 to 2019) and Trends of Filled Opioid and Nonopioid Prescriptions Within 30 Days After Total Knee Arthroplasty

Year	Opioid		Nonopioid	
	n	%	n	%
2010	37,546	45.31%	5,561	6.71%
2011	30,393	52.64%	5,383	9.32%
2012	28,199	53.53%	5,407	10.26%
2013	29,489	56.89%	6,271	12.10%
2014	27,611	59.04%	6,455	13.80%
2015	29,178	59.64%	7,504	15.34%
2016	38,777	60.81%	11,078	17.37%
2017	34,458	59.96%	10,700	18.62%
2018	34,361	60.24%	12,141	21.29%
2019	37,276	61.95%	14,766	24.54%
*P*-value				
2010-2015		**0.004**		**<0.001**
2016-2019		0.489		**0.018**
2010-2019		**<0.001**		**<0.001**
CAGR				
2010-2015		**5.65%**		**17.98%**
2016-2019		0.62%		**12.21%**
2010-2019		**3.54%**		**15.50%**

*P* values <0.05 bolded for significance and correlating compound annual growth rates (CAGRs).

Trends in the average daily MME significantly decreased between all time points: 2010 to 2015 (*P* = 0.038; CAGR = −4.44%), 2016 to 2019 (*P* < 0.001; CAGR = −5.23%), and 2010 to 2019 (*P* < 0.001; CAGR = −4.21%) (Table 2).

**Table 2:** Daily Average Milligram Morphine Equivalents and Trends From 2010 to 2019 of Filled Prescriptions Within 30 Days After Total Knee Arthroplasty

Year	MME	SD
2010	55.06	46.37
2011	46.98	41.36
2012	45.78	42.17
2013	44.71	39.82
2014	43.85	38.13
2015	43.87	39.12
2016	43.93	41.24
2017	41.86	37.63
2018	39.61	37.61
2019	37.39	33.17
*P*-value		
2010-2015	**0.038**	**0.021**
2016-2019	**<0.001**	0.052
2010-2019	**<0.001**	**0.001**
CAGR		
2010-2015	**−4.44%**	**−3.34%**
2016-2019	**−5.23%**	−7.00%
2010-2019	**−4.21%**	**−3.65%**

MME = milligram morphine equivalent

*P* values <0.05 bolded for significance and correlating compound annual growth rates (CAGRs).

### 90-Day Prescription Claim Rates

**Figure 3:**
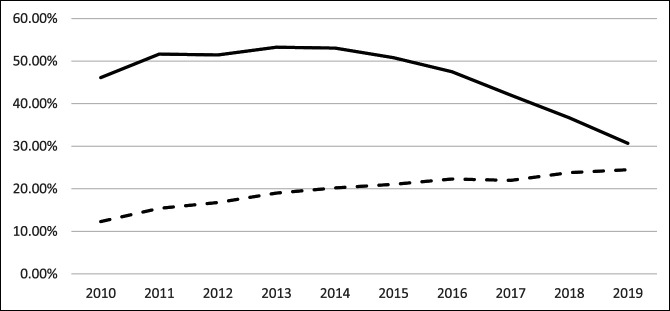
Graph showing annual rates of filled prescriptions for opioids and nonopioids within 90 days of TKA from 2010 to 2019. TKA = total knee arthroplasty

Nonopioid prescriptions increased significantly from 2010 to 2015 (*P* < 0.001; CAGR = 11.34%) while trends for opioids were not significant (*P* = 0.212). Trends between 2016 and 2019 for opioids significantly decreased (*P* < 0.001; CAGR = −13.53%) while trends for nonopioids did not show significance. Overall trends between 2010 and 2019 were significant for decreasing rates of opioids (*P* = 0.013; CAGR = −4.42) and increasing rates of nonopioids (*P* < 0.001; CAGR = 7.95%) (Table 3, Figure 3).

**Table 3:** Annual Prevalence (2010 to 2019) and Trends of Filled Opioid and Nonopioid Prescriptions Within 90 Days After Total Knee Arthroplasty

Year	Opioid		Nonopioid	
	n	%	n	%
2010	38,246	46.15%	10,194	12.30%
2011	29,818	51.64%	8,892	15.40%
2012	27,111	51.46%	8,848	16.80%
2013	27,623	53.29%	9,852	19.01%
2014	24,815	53.06%	9,452	20.21%
2015	24,865	50.83%	10,297	21.05%
2016	30,290	47.50%	14,239	22.33%
2017	24,151	42.02%	12,631	21.98%
2018	20,940	36.71%	13,573	23.80%
2019	18,480	30.71%	14,737	24.49%
*P*-value				
2010-2015		0.212		**<0.001**
2016-2019		**<0.001**		0.103
2010-2019		**0.013**		**<0.001**
CAGR				
2010-2015		1.95%		**11.34%**
2016-2019		**−13.53%**		3.13%
2010-2019		**−4.42%**		**7.95%**

*P* values <0.05 bolded for significance and correlating compound annual growth rates (CAGRs).

Average daily MME significantly decreased across all time points: 2010 to 2015 (*P* = 0.038; CAGR = −4.44%), 2016 to 2019 (*P* < 0.001; CAGR = −5.26%), and 2010 to 2019 (*P* < 0.001; CAGR = −4.22%) (Table 4).

**Table 4:** Daily Average Milligram Morphine Equivalents and Trends From 2010 to 2019 of Filled Prescriptions Within 90 Days After Total Knee Arthroplasty

Year	MME	SD
2010	55.13	46.44
2011	47.04	41.45
2012	45.82	42.26
2013	44.75	39.9
2014	43.88	38.18
2015	43.92	39.2
2016	43.98	41.31
2017	41.91	37.71
2018	39.85	37.69
2019	37.40	33.23
*P*-value		
2010-2015	**0.038**	**0.021**
2016-2019	**<0.001**	0.052
2010-2019	**<0.001**	**0.001**
CAGR		
2010-2015	**−4.44%**	**−3.33%**
2016-2019	**−5.26%**	−7.00%
2010-2019	**−4.22%**	**−3.65%**

MME = milligram morphine equivalent

*P* values <0.05 bolded for significance and correlating compound annual growth rates (CAGRs).

### 1-Year Prescription Claim Rates

**Figure 4:**
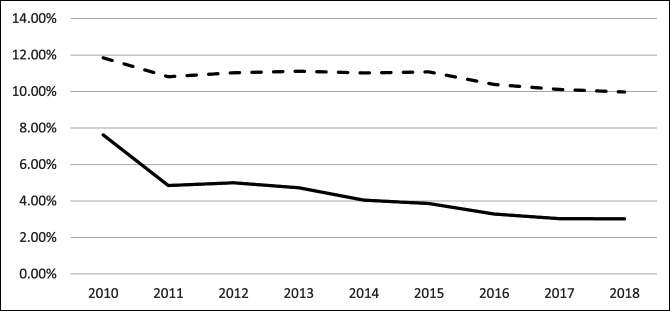
Graph showing annual rates of filled prescriptions for opioids and nonopioids within 1 year of TKA from 2010 to 2018. TKA = total knee arthroplasty

Trends for opioid prescriptions from 2010 to 2015 significantly decreased (*P* = 0.032; CAGR = −12.72%) while nonopioid trends were not significant (*P* = 0.320). Neither opioids (*P* = 0.316) nor nonopioid (*P* = 0.121) trends were significant from 2016 to 2019. Trends from 2010 to 2019 significantly decreased for both opioids (*P* = 0.001; CAGR = −10.92%) and nonopioids (*P* = 0.003; CAGR = −2.12%) (Table 5, Figure 4).

**Table 5:** Annual Prevalence (2010 to 2019) and Trends of Filled Opioid and Nonopioid Prescriptions Within 1 Year After Total Knee Arthroplasty

Year	Opioid		Nonopioid	
	n	%	n	%
2010	6,317	7.62%	9,820	11.85%
2011	2,802	4.85%	6,243	10.81%
2012	2,633	5.00%	5,810	11.03%
2013	2,449	4.72%	5,761	11.11%
2014	1,893	4.05%	5,153	11.02%
2015	1,889	3.86%	5,419	11.08%
2016	2,097	3.29%	6,624	10.39%
2017	1,741	3.03%	5,812	10.11%
2018	1,724	3.02%	5,692	9.98%
*P*-value				
2010-2015		**0.032**		0.320
2016-2019		0.316		0.121
2010-2019		**0.001**		**0.003**
CAGR				
2010-2015		**−12.72%**		−1.34%
2016-2019		−4.13%		−1.99%
2010-2019		**−10.92%**		**−2.12%**

*P* values <0.05 bolded for significance and correlating compound annual growth rates (CAGRs).

## Discussion

Using a national claims database, this study found varying trends in postoperative TKA opioid utilization from 2010 to 2019. The PearlDiver insurance database allowed for selection of anonymized patient data based on history of psychiatric diagnoses, prescription medication history, and mental health history (Figure 1), which enabled better examination of the risk of prolonged use of opioids after TKA. Between 2010 and 2019, three distinct time frames were analyzed after TKA: 30 days, 90 days, and 1 year postoperatively. At 30 days postoperatively, average opioid prescriptions increased until 2015 before flattening while nonopioids prescriptions continued to increase across all years analyzed. Ninety-day opioid prescriptions showed no notable trends from 2010 to 2015 but decreased markedly after 2015. For the same 90-day postoperative period, nonopioid prescriptions increased from 2010 to 2015 and then flattened. At 1 year postoperatively, average opioid prescriptions decreased leading up to 2016 before leveling out; a similar pattern was observed in nonopioid prescriptions.

The linear regression of MME at 30 days postoperatively annually was 0.35% less than that of the linear regression of MME at 90 days postoperatively annually, indicating the reduction in opioids was relatively consistent from 30 to 90 days postoperatively. This is possibly explained by stable pain trajectories after TKA were seen in the same 30- and 90-day periods in an analysis by Dumenci et al,^14^ particularly in patients with poor surgical outcomes.

At 30 days postoperatively, the rate of opioid prescriptions increased until 2015 and then plateaued while nonopioid prescriptions continued to increase. This timing demonstrates that the change in American Academy of Orthopaedic Surgeons protocol may have affected physician prescribing behaviors. Two studies by Reid et al analyzed data from 2016 to 2017 on the impact of state-level legislation in Rhode Island on limiting opioid distribution after orthopaedic procedures and found notable year-over-year reduction in initial and 30-day postoperative opioid prescriptions after such laws went into place after spine surgery and a >50% decrease in the number of opioid pills prescribed after six common orthopaedic procedures.^15,16^ This varies from our findings that show a plateau in opioid prescriptions was reduced after 2016 rather than a year-over-year decrease in rates of opioid prescriptions. One likely explanation for the discrepant data could be because of the small population of Rhode Island, and its effect was negligible given this study's nationwide database used. Regardless, legislation alike also played a role in opioid prescriptions over the study period.

When examining opioid prescriptions at 90 days (Table 3), no clear trend was observed through 2015; however, prescriptions starting in 2016 decreased precipitously in what ended up being a change from 46.15% of patients filling prescriptions at 90 days postoperatively to 30.71%. Conversely, nonopioid prescriptions increased steadily from 2010 to 2016 and then plateaued (Figure 3). This divergence means that a certain number of patients were no longer filling prescriptions for any pain medications—opioid or nonopioid. This finding is noteworthy because it may signify overprescribing of opioids after TKA, which is supported by a study by Etcheson et al^17^ who found no association between total opioid consumption after TKA and factors like patient's pain management satisfaction or overall hospital rating.

At 1 year postoperative opioid prescriptions decreased before 2016 and then flattened. For this period, both nonopioid and opioid prescription claims leveled out after 2016. Approximately 3% of TKA patients who were prescribed opioids continued to refill their prescription at and beyond 1 year. This differs from findings in a study by Cook et al,^18^ where 13% of TKA patients with no history of opioid use 6 months before surgery continued to fill prescriptions 1 year after surgery. Of course, there are other factors that influence postoperative pain for which data were not examined in our retrospective study. Among these factors is surgical time, where longer tourniquet time has previously been correlated with persistent opioid use after TKA.^19^ Other factors include sex, age (at the time of surgery), mental health history, and preoperative opioid use.^20^

There are many variables that affect patient experience and outcome, and guidelines and regulations were not implemented in a vacuum. With the increased profile of opioids addictive chemistry, perioperative anesthesia techniques (regional and spinal anesthesia) and multimodal pain management have received growing support in multiple areas of medicine. A 2014 randomized controlled trial by Lamplot et al studying multimodal pain management in TKA patients found that the multimodal approach which targeted peripheral and central pathways resulted in notable improvements in patients' satisfaction and speed of rehabilitation while at the same time decreasing utilization of opioids.^21^ This study contributes evidence toward a movement promoting multimodal pain management as the standard of care, in turn reducing dependence on opioids.

This examination of insurance claims data relies on provider coding and proper billing habits of institutions. The database cannot be used to determine from which provider the prescriptions originated from. While prescriptions filled less than 30 days after TKA were more likely to be prescribed by an orthopaedic provider, this could not be ascertained from PearlDiver. Another limitation is that many nonopioids can be bought over the counter and could not be assessed because only prescribed medications would show up in the PearlDiver database. Pain was also not able to be assessed in this analysis. Regardless, the data provided helps assess prescription practices after TKA. While the data presented here does not include dose, drug, prescription length, or consumption of opioids by patients, it does highlight the decreasing volume of circulating opioids after TKA.

## Conclusion

This study's analysis found a trend of reduced MME filled after TKA within 30 and 90 days postoperatively. Current trends of opioid prescriptions filled are stable at 30 days and decreasing at 90 days and 1 year. Nonopioids are also being increasingly used within 30 days of TKA while chronic use of both opioids and nonopioids after TKA are reducing greatly. While these findings are impactful to the opioid epidemic, there are limitations to what these patterns can be attributed to. Factors such as societal or governmental guidelines, physician awareness about the opioid epidemic and the resulting changes in prescribing behavior, and local legislation on prescribing opioids influence prescriptions over the period studied. Additional analysis, while accounting for these factors, is needed to determine which methods had the greatest effect on combating the opioid epidemic.

## Supplementary Material
