# Supplementary material for: Trends From 2010 to 2019 in Opioid and Nonopioid Pain Management After Total Knee Arthroplasty
Source: J Am Acad Orthop Surg Glob Res Rev. 2024 Jun 12;8(6):e23.00062. doi: 10.5435/JAAOSGlobal-D-23-00062 (PMC11175854; doi:10.5435/JAAOSGlobal-D-23-00062)
Supplement: Supplementary file 1 [file jagrr-8-e23.00062-s001.docx]

Supplemental Appendix

| **Cohort Criteria** | |
| --- | --- |
| Total Knee Arthroplasty | CPT-27447, ICD-10-P-0SRC069, ICD-10-P-0SRC06A, ICD-10-P-0SRC06Z, ICD-10-P-0SRC0J9, ICD-10-P-0SRC0JA, ICD-10-P-0SRC0JZ, ICD-10-P-0SRC0LA, ICD-10-P-0SRC0LZ, ICD-10-P-0SRC0L9, ICD-10-P-0SRC0M9, ICD-10-P-0SRC0MA, ICD-10-P-0SRC0MZ, ICD-10-P-0SRC0NA, ICD-10-P-0SRC0NZ, ICD-10-P-0SRC0N9, ICD-10-P-0SRD069, ICD-10-P-0SRD06A, ICD-10-P-0SRD06Z, ICD-10-P-0SRD0J9, ICD-10-P-0SRD0JA, ICD-10-P-0SRD0JZ, ICD-10-P-0SRD0LA, ICD-10-P-0SRD0LZ, ICD-10-P-0SRD0L9, ICD-10-P-0SRD0M9, ICD-10-P-0SRD0MA, ICD-10-P-0SRD0MZ, ICD-10-P-0SRD0NA, ICD-10-P-0SRD0NZ, ICD-10-P-0SRD0N9, ICD-9-D-8154 |
| Exclusion Criteria - (mood disorders, personality disorders, psychotic disorders, psychosis, dementia, substance abuse, substance dependance, fibromyalgia, neuralgia, complex regional pain syndrome, central pain syndrome, neoplastic pain, chronic pain) | ICD-10-D-M797, ICD-9-D-7291, ICD-10-D-F320:ICD-10-D-F339, ICD-9-D-29620:ICD-9-D-29636, ICD-10-D-M792, ICD-9-D-7292, ICD-10-D-B0222, ICD-9-D-05312, ICD-9-D-05313 , ICD-10-D-G500, ICD-9-D-3501, ICD-9-D-3380, ICD-9-D-33821, ICD-9-D-33822, ICD-9-D-33828, ICD-9-D-33829, ICD-9-D-3384, ICD-9-D-3383, ICD-9-D-33720, ICD-9-D-33721, ICD-9-D-33722, ICD-9-D-33729, ICD-10-D-G890, ICD-10-D-G8921, ICD-10-D-G8922, ICD-10-D-G8928, ICD-10-D-G8929, ICD-10-D-G893, ICD-10-D-G894, ICD-10-D-G9050, ICD-10-D-G90511, ICD-10-D-G90512, ICD-10-D-G90513, ICD-10-D-G90519, ICD-10-D-G90521, ICD-10-D-G90522, ICD-10-D-G90523, ICD-10-D-G90529, ICD-10-D-G9059, ICD-9-D-30780, ICD-9-D-30789, ICD-10-D-F4541, ICD-10-D-F4542, ICD-9-D-29383, ICD-10-D-F0630: ICD-10-D-F0634, ICD-9-D-29690, ICD-10-D-F39, ICD-9-D-29699, ICD-10-D-F348, ICD-9-D-29383, ICD-9-D-29384, ICD-10-D-F064, ICD-9-D-30000, ICD-10-D- F064, ICD-9-D-30002, ICD-10-D-F064, ICD-9-D-30009, ICD-10-D-F413, ICD-10-D-F418, ICD-9-D-29600: ICD-9-D-29616, ICD-9-D-29640: ICD-9-D-29689, ICD-10-D-F3010: ICD-10-D-F319, ICD-9-D-2900: ICD-9-D-29043, ICD-10-D-F0150: ICD-10-D-F0391, ICD-10-D-F05, ICD-10-D-F1027, ICD-9-D-2912, ICD-10-D-F1997, ICD-9-D-29282, ICD-9-D-29410: ICD-9-D-29421, ICD-10-D-G3109, ICD-9-D-33119, ICD-9-D-33182, ICD-10-D-G3183, ICD-9-D-29381, ICD-10-D-F062, ICD-9-D-29382, ICD-10-D-F060, ICD-9-D-2908, ICD-10-D-F0390, ICD-9-D-2909, ICD-9-D-29010: ICD-9-D-29021, ICD-9-D-2973, ICD-10-D-F24, ICD-9-D-2980, ICD-9-D-2981, ICD-10-D-F323, ICD-10-D-F333, ICD-9-D-29634, ICD-9-D-2984, ICD-10-D-F23, ICD-9-D-2988, ICD-9-D-2989, ICD-10-D-F29, ICD-9-D-3010: ICD-9-D-3019, ICD-10-D-F600: ICD-10-D-F609, ICD-10-D-F6810: ICD-10-D-F6813, ICD-9-D-29500: ICD-9-D-29595, ICD-10-D-F200: ICD-10-D-F21, ICD-10-D-F250: ICD-10-D-F259, ICD-9-D-V110, ICD-10-D-F601, ICD-9-D-30120, ICD-9-D-30122, ICD-9-D-30390: ICD-9-D-30493, ICD-10-D-F1010: ICD-10-D-F1999 |
| **Drug Codes** | |
| Opioids | USC-02211, USC-02212, USC-02214, USC-02221, USC-02222, USC-02232 |
| Gabapentinoids | GENERIC_DRUG-PREGABALIN, DRUG-LYRICA, GENERIC_DRUG-GABAPENTIN, DRUG-GRALISE, DRUG-HORIZANT, DRUG-NEURONTIN, DRUG-GABAPENTIN, DRUG-PREGABALIN |
| Nonsteroidal Anti-inflammatory | GENERIC_DRUG-KETOROLAC_TROMETHAMINE, GENERIC_DRUG-KETOROLAC_TROMETHAMINE/PF, GENERIC_DRUG-CELECOXIB, DRUG-CELEBREX, DRUG-ADVIL, DRUG-ANAPROX, DRUG-ANAPROX_DS, DRUG-MOTRIN, DRUG-ALEVE, GENERIC_DRUG-IBUPROFEN, GENERIC_DRUG-NAPROXEN, DRUG-ACUVAIL, DRUG-ACULAR, DRUG-ACULAR_LS, DRUG-NAPROSYN, DRUG-EC-NAPROSYN, DRUG-WAL-PROXEN, GENERIC_DRUG-MELOXICAM, DRUG-MOBIC, DRUG-VIVLODEX,GENERIC_DRUG-DICLOFENAC_SODIUM, GENERIC_DRUG-DICLOFENAC_POTASSIUM, DRUG-CAMBIA, DRUG-CATAFLAM, DRUG-VOLTAREN, DRUG-VOLTAREN-XR DRUG-ZORVOLEX |
|  |  |
|  |  |
